# Effect of e-liquid flavor on electronic cigarette topography and consumption behavior in a 2-week natural environment switching study

**DOI:** 10.1371/journal.pone.0196640

**Published:** 2018-05-02

**Authors:** R. J. Robinson, E. C. Hensel, A. A. al-Olayan, J. M. Nonnemaker, Y. O. Lee

**Affiliations:** 1 Department of Mechanical Engineering, Rochester Institute of Technology, Rochester, NY, United States of America; 2 Research Triangle International, Inc., Research Triangle Park, NC, United States of America; Brown University, UNITED STATES

## Abstract

Electronic Nicotine Delivery Systems (ENDS) offer an alternate means to consume nicotine in a variety of flavored aerosols. Data are needed to better understand the impact of flavors on use behavior. A natural environment observational study was conducted on experienced ENDS users to measure the effect of e-liquid flavor on topography and consumption behavior. The RIT wPUM^TM^ monitor was used to record to record the date and time and puff topography (flow rate, volume, duration) for every puff taken by N = 34 participants over the course of two weeks. All participants used tobacco flavor for one week, and either berry or menthol flavor for one week. Results provide strong evidence that flavor affects the topography behaviors of mean puff flow rate and mean puff volume, and there is insufficient evidence to support an influence of flavor on mean puff duration and mean puff interval. There was insufficient evidence, due to the low power associated with the limited number of observation days, to establish a relationship between flavor and cumulative consumption behavior. While the results indicate that an effect may be evident, additional observation days are required to establish significance.

## Introduction

### Background and rationale

The 2009 Family Smoking Prevention and Control Act and 2016 Deeming Rule, gives the Food and Drug Administration (FDA) authority to regulate products including ENDS [1–3]. The FDA recognizes that regulation must be informed in-part by scientific findings on use behavior, including topography and consumption behavior associated with specific products and product components [4–6]. While cigarette smoking behavior has been widely studied [7–17],emerging evidence suggests that topography and consumption behavior associated with ENDS use differs from cigarette smoking [18–20]. Meaningful risk assessment associated with ENDS use is hindered by the wide variation in types of ENDS and e-liquid flavors available.

The emerging literature on e-liquid flavor additives support the premise that flavor is an important factor in e-cigarette use [21–24] and some flavors, such as menthol, have known analgesic and sensory effects present in cigarettes and other tobacco products [25], while others have wide appeal such as sweet/fruity flavors [22] [26]. E-cigarette users and cigarette smokers report flavor and related sensory characteristics as reasons for using and valuing e-cigarettes [21, 22, 27, 28]. Consistent with this, e-cigarette companies create and promote over 15,000 of flavors for consumers in the marketplace, predominantly in tobacco, menthol, alcohol/drink, fruit, and dessert/candy flavors [29]. Flavors, particularly menthol [30], may influence topography. It is currently unknown whether different flavors of e-cigarettes are associated with varying topography patterns. If so, then some flavors could result in topographies that increase exposure to nicotine and other harmful or potentially harmful e-cigarette constituents. Yet knowledge on topography and consumption as a function e-liquid flavor is lacking.

Several studies provide ENDS use topography [18, 31–35] based on laboratory environment measurements, but none address how e-liquid flavors influence user topography and consumption behavior. Differences between laboratory and natural environment topography and consumption have been demonstrated for cigarettes [36], suggesting that use behavior is best assessed in the naturalistic setting. Natural environment studies have been done for ENDS [37, 38], but no naturalistic study has examined the impact of e-liquid flavor on use behavior.

Herein, we present data from a two-week flavor switching study and provide results with adequate statistical power to make inferences regarding topography characteristics as a function of e-liquid flavor. We employ a natural use environment which has the advantage of not interfering with normal usage patterns [39] and employ monitoring protocol as previously demonstrated [40, 41]. Study results provide quantitative data demonstrating an impact of e-liquid flavor on topography behavior. The study informs regulatory policy regarding Premarket Tobacco Product Applications for Electronic Nicotine Delivery Systems (81 FR 28781) and supports the need to develop meaningful product and component specific protocols to test ENDS and ENDS components.

### Objectives

The goal of the study was to evaluate the impact of e-liquid flavor on topography behavior, including puff flow rate, puff volume, puff duration and puff interval and consumption behavior, including mean daily puffs, mean daily volume, and cumulative weekly volume among experienced ENDS.

## Methods

### Study design

The study was designed to test the hypothesis that participants would alter their puff topography and total consumption when switching between e-liquid flavors. The hypothesis was tested in a two-week observational on experienced pen-style e-cig users’. Study participants used their preferred nicotine concentration and vaped at their desired usage patterns for the entire two-week period in their natural environment. The e-liquid flavors used in this study were tobacco, menthol, and berry. We selected tobacco flavor because it is regularly used as a comparison condition in flavor studies of e-cigarettes [23, 24], menthol flavor because of the established literature on the sensory properties of menthol [25] and berry flavor because of the appeal of sweet/fruity flavored e-cigarette liquids [22][26]. Participants were assigned one e-liquid flavor for the first week and a second e-liquid flavor for the second week. Participants recorded every vaping session with RIT’s wPUM^TM^ topography and use behavior monitor. The study was designed to facilitate a pairwise comparison of topography and consumption behavior for usage of tobacco flavor compared to either berry or menthol flavor. Flavor assignments were randomized and balanced by participant and by order of assignment. The groups are shown in Table 1. The study purpose and protocol were reviewed and approved by the Rochester Institute of Technology (RIT) Human Subjects Research Office Institutional Review Board (IRB) and the RTI International IRB.

**Table 1:** Study design.

Flavor Groups	Protocol	Week 1 Flavor Assignment	Week 2 Flavor Assignment
T/M	C1	Tobacco	Menthol
	C2	Menthol	Tobacco
T/B	C3	Tobacco	Berry
	C4	Berry	Tobacco

The study included four randomized groups. C1 and C2 are referred to as the Tobacco/Menthol (T/M) group. C3 and C4 are referred to as the Tobacco/Berry (T/B) group. Switching order was randomized and balanced in each group.

### Natural environment monitoring protocol

#### 1. Recruitment

The target population was the RIT campus community which consists of approximately 15,400 undergraduate students, 3,200 graduate students, and 3,800 faculty and staff. The student population includes approximately 1,200 deaf and hard-of-hearing students. Participants were recruited using mass-emails sent to the campus distribution list in conjunction with flyers posted around the campus between July and October of 2016. Both the mass-email and the flyers advertised a research study regarding electronic cigarettes and stated that participants may be eligible to receive $300 for participating in a 14-day study, if they were between the ages of 18 and 65 and had been e-cig users for at least 3 months. Anyone interested was asked to contact the research administrator at the email provided.

#### 2. Pre-screening

The research administrator responded to each email received from a potential participant, sending a reply email with detailed information about the study and a link to a pre-screening questionnaire intended to identify and exclude individuals who did not meet the eligibility requirements. Individuals passed the pre-screening if their responses indicated that they consented to participate, were between 18–65 years of age, and were regular users of the pen-style e-cig or a device similar to the NJOY^TM^ device which they would be asked to use in the study. Individuals were considered regular Electronic Vape Pen (EVP) users if they identified that (i) they vaped weekly for the past 3 months, (ii) they used EVP with bottled liquid and no heat adjustment 4–7 of the last 7 days, (iii) the most common EVP they used was with bottled liquid and no heat adjustment, and (iv) they vaped with nicotine either every day or some days. Interested participants must have also provided the nicotine strength of their choice e-liquid when prompted.

Individuals who did not pass the pre-screening were notified immediately after taking the on-line survey. Others were invited to schedule an intake appointment at the Respiratory Technologies Lab (RTL) at RIT.

#### 3. Pre-Deployment wPUM^TM^ monitor calibration

**Fig 1:**
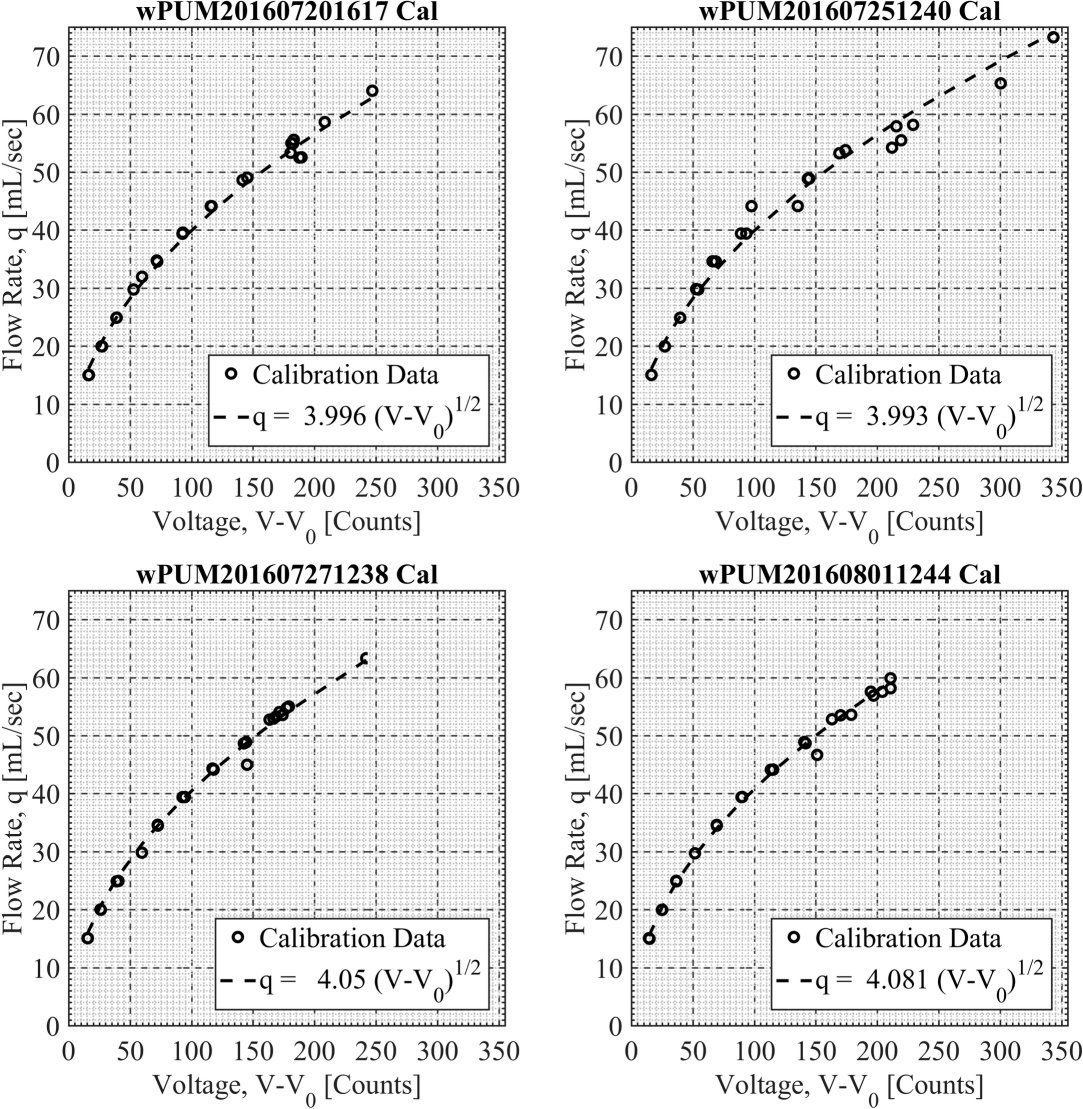
Exemplar wPUM^TM^ monitor calibration data obtained from the PES^TM^-1 calibration system. Shown are the pre-deployment week-1 (20160720), mid-week-1 (20160725), pre-deployment week-2 (20160727), and mid-week-2 (20160801) calibration results for wPUM^TM^ monitor 3 used by participant 3. These calibration data document wPUM^TM^ monitor-specific performance and the potential impact of monitor contamination during natural use monitoring. Effects are mitigated by pre- and post- calibration protocols and TAP^TM^ program signal processing algorithms. Underlying data is available in S1, S2, S3, S4 Datasets.

Prior to the participant’s intake appointment, a technician conducted a pre-deployment flow rate calibration of the wPUM^TM^ monitor anticipated for assignment to that participant. Flow rate calibration was done using the fully characterized RIT PES-1^TM^ calibration system, which employs flow meters certified annually by a third-party vendor. Each calibration resulted in a calibration curve relating individual wPUM^TM^ monitoring device voltage readings to the primary instrument flow rate measurement. Exemplar wPUM^TM^ monitor calibration curves are shown in **Fig 1**.

It should be noted that the RIT calibration protocol utilized in this study differed from that described in the PhenX protocol supplemental information for “Puffing Profile” using the CReSS^TM^ device. The PhenX procedure does not describe a thorough flow rate calibration, but rather describes a repeatability test: “The device is calibrated by taking 5 consecutive draws on the syringe at each of three levels: 20 mL, 35 mL, and 50 mL. Devices must yield values within 3 mL of each draw to pass calibration.” Flow rate calibration for this study is conducted by correlating wPUM^TM^ monitor readings to an independent flow rate measurement source using repeated measures across the full range of flow rates anticipated for the EVP. Each monitor was calibrated prior to each weekly flavor change, and during a mid-week maintenance check.

#### 4. Intake appointment

Intake appointments took place in the RTL on Wednesdays of each week, and were made on a first-come, first-serve basis. Each appointment lasted between fifteen minutes to one hour, and included final screening via on-line questionnaire, verification of age by government-issued identification, informed consent, confirmation of the participant’s preferred nicotine level, preparation of the pre-filled e-liquid tank with the assigned week-1 flavor, and training on the wPUM^TM^ monitor. Participants who were excluded during the intake appointment were given $5 cash. Participants who signed the informed consent were provided a pre-filled e-liquid tank with assigned week-1 flavor at the participant’s preferred nicotine level, an NJOY^TM^ pen-style e-cig, the pre-calibrated wPUM^TM^ monitor and a study packet describing the study protocol and monitor operation. Participants were instructed on the proper use of the monitor and given an opportunity to turn it on and off in the lab. Participants were invited to contact the research administrator during the observation period if they encountered any difficulties. Participants were asked to work by email with the research administrator to schedule the five remaining in-lab appointments, and then dismissed.

#### 5. Monitoring period

The monitoring period began immediately after the intake appointment and lasted for two weeks. The study protocol was designed to begin and end on a Wednesday to capture weekday and weekend behavior without interruption. Participants were instructed to vape naturally in their own environment, using the NJOY^TM^ vape pen and pre-filled tank provided to them along with the wPUM^TM^ monitor for every vaping session. At their intake appointment, participants were provided tanks containing their assigned week-1 flavor and their preferred nicotine concentration to use for the first week. At their switching appointment, on Wednesday ending the first week, participants were provided tanks containing their assigned week-2 flavor and their preferred (same as week 1) nicotine concentration to use for the second week. Before concluding the switching appointment, the week-1 tanks were collected and weighed, and a monitor calibration and maintenance check was done. Participants used their week-2 tanks for one week and returned to the RTL on Wednesday ending the second week for their outtake appointment.

Participants were also asked to bring the monitor, EVP and e-liquid vials to the lab mid-way through each monitoring week for a calibration and maintenance check. During the calibration and maintenance check, monitors were checked for issues such as battery drain, condensation or cracked casing. The administrator verified each participant had sufficient e-liquid supplies. Monitors were cleaned and re-calibrated, and replaced as needed so the participant could continue the study uninterrupted. Participants were also invited to stop by the lab any time during their observation period if they had questions or problems with the monitor, the NJOY^TM^ EVP or the assigned e-liquid.

#### 6. Outtake appointment

Outtake appointments took place in the RTL. Participants returned the wPUM^TM^ monitor, vape pens and tank with unused e-liquid to the RTL. The research administrator conducted an exit interview to verify product and monitor use during the observation period and identify difficulties encountered during the study.

#### 7. Post-deployment wPUM^TM^ monitor calibration

Post-deployment calibrations were conducted on each wPUM^TM^ monitor to determine if the monitor had been affected by condensation, or fluid or particulate build-up and account for these changes in monitor performance to confirm the accuracy of the recorded flow rates. The post-deployment calibration curve was compared to the pre-deployment and mid-week calibration curves, and if changes were observed, the analyst adjusted parameters in the TAP^TM^ program accordingly to assess the impact of the change on the topography characteristic obtained from the TAP^TM^. This critical step allows us to accurately convert the raw monitoring voltage to flow rate measurements and to put error bounds around assessed puff flow rates and puff volumes which reflect observed variation in the calibrated monitor performance.

### Data analysis

Following each monitoring period, data was analyzed in four phases: data integrity management (phase 0), puff identification (phase 1), descriptive statistics (phase 2) and inferential statistics (phase 3).

**Fig 2:**
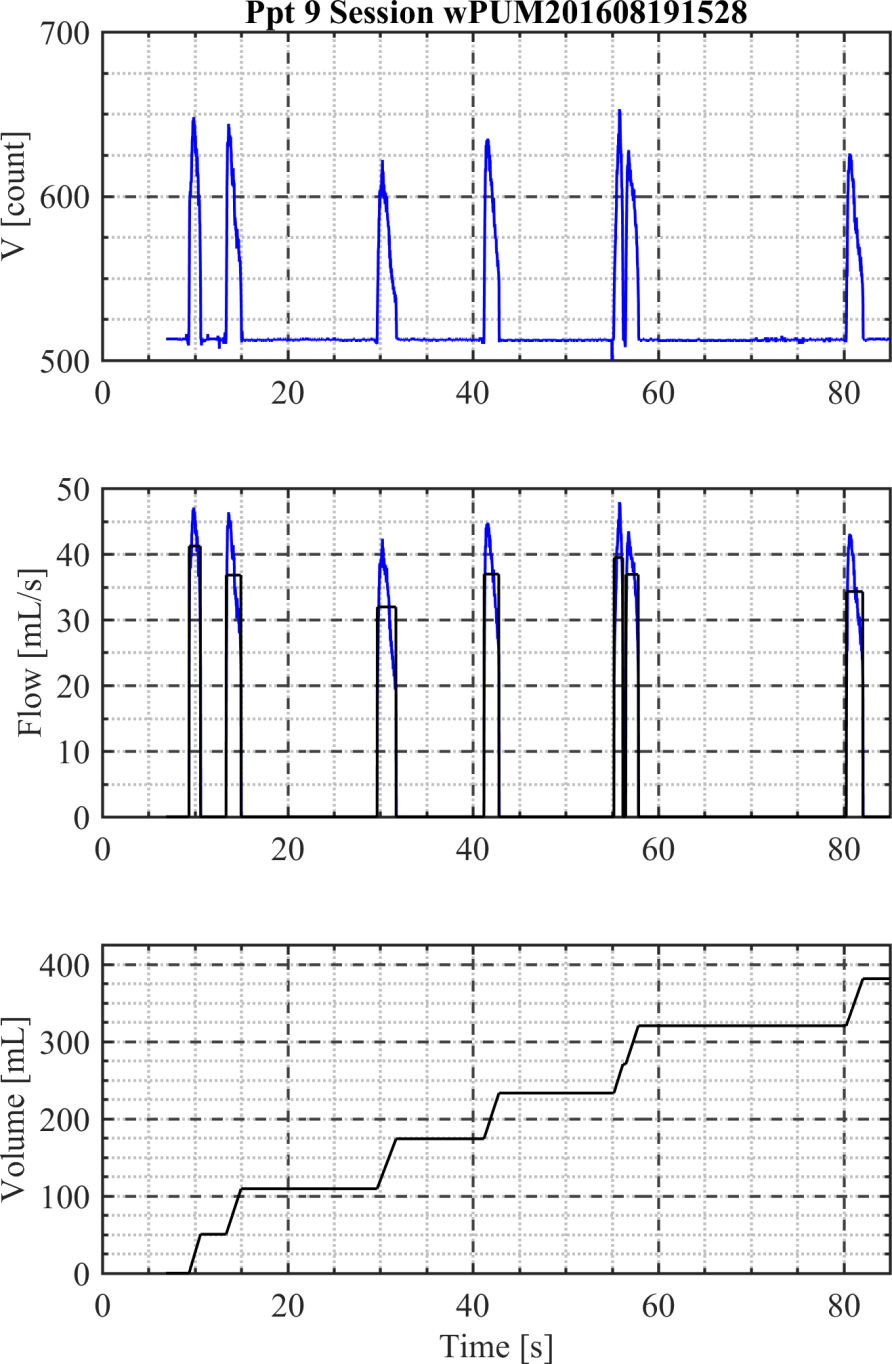
Exemplar topography and consumption behavior for a single vaping session. Shown is an example resulting from phase 1 of the analysis. The TAP^TM^ program converts raw noisy monitoring data (top panel) into discrete identifiable puffs with known flow rate (middle panel). Cumulative session volume (bottom panel) is determined by summing the individual puff volumes. Phase 1 results in session topography with puffs of known duration, mean flow rate, puff volume and inter-puff interval. Underlying data is available in S5 Dataset.

In phase 0, each voltage file captured with the wPUM^TM^ monitor was inspected by a data analyst for the presence of any unusual characteristics which might have impeded subsequent data analysis. For example, the analyst looked for signal drift which may have been caused by contamination of the pressure sensor or inconsistent usage patterns indicating the participant failed to turn the monitor on or off. The analyst made judgement calls and documented their assessment of the raw monitoring data in an “analysis protocol file” for each participant, which described the numerical process to be completed for each session of each participant. The “analysis protocol file” was preserved in a version controlled secured repository and provides an audit trail of processing conducted on each data file of each participant. In phase 1, the analyst ran the TAP^TM^ program which read the noisy raw monitoring voltage data, and identified discrete puffs by identifying the starting and ending time of each puff. The TAP^TM^ program applied the calibration curves (**Fig 1**) to covert the raw voltage measurements into flow rate data. Knowing the puff duration and transient flow rates for each puff, the TAP^TM^ program determined the mean puff flow rate, puff volume and inter-puff interval for each puff. The cumulative volume and puff count were determined for each session, each day, and each flavor condition over the monitoring period for each participant. Fig 2 illustrates the puff identification process resulting from the TAP^TM^ topography code for one exemplar puffing session of Participant 9.

The upper panel of Fig 2 shows the time history of the voltage data recorded by the monitor, which is converted to the transient flow rate using the monitor-specific calibration curve in the middle panel. The transient flow rate date during each puff is analyzed as the equivalent duration with a mean puff flow rate yielding the same integrated puff volume, shown in the lower panel, as the dynamic flow measurements. In phase 2, the TAP^TM^ program was used to compute topography behavior characteristics including minimum, maximum and mean values for puff flow rate, puff volume, puff duration and interpuff interval along with the standard deviation and the 95% confidence intervals on the means, per puffing session, per day, and per flavor condition for each participant for each topography behavior indicator. The TAP^TM^ program was used to compute consumption behavior characteristics including mean daily volume, mean daily puff count, cumulative weekly volume, and cumulative weekly puff count, along with the standard deviation and the 95% confidence intervals on the means, per flavor condition for each participant. Mean topography behavior characteristics for each participant were computed by taking the sum over the entire observation period and dividing by the total number of puffs. Mean daily consumption behavior characteristics for each participant were computed by first computing a daily average (total divided by number of puffs that day), then summing the daily averages over the observation period and dividing by the number of days in the observation period. Descriptive statistics by participant and by flavor condition are provided in the supplemental data.

In phase 3, the TAP^TM^ program was used to test several inferences related to the impact of flavor on topography and consumption behavior. The study design was verified using a between groups ANOVA to test the impact of switching order on the outcome measures (*e*.*g*., C1 *vs*. v2 and C3 *vs*. c4). A pair-wise comparison of topography and consumption behavior indicators was conducted for each participant as they switched between flavors. A Bonferroni correction factor (α_c_ = 0.00147) was applied to each pair-wise comparison to compensate for the Family Wise Error Rate (FWER) at α = 0.05 significance level. Each two-sided *t*-test was conducted using the assumptions that each week of observations are from normal distributions with unknown and unequal variances, the Behrens-Fisher problem, using Satterthwaite's approximation for the effective number of degrees of freedom. Topography behavior indicators included mean puff flow rate, mean puff duration, mean puff volume, and mean puff interval. Consumption behavior indicators included mean daily volume, mean daily puff count, cumulative volume per condition, and cumulative puff count per condition.

## Results

### Study cohort

**Fig 3:**
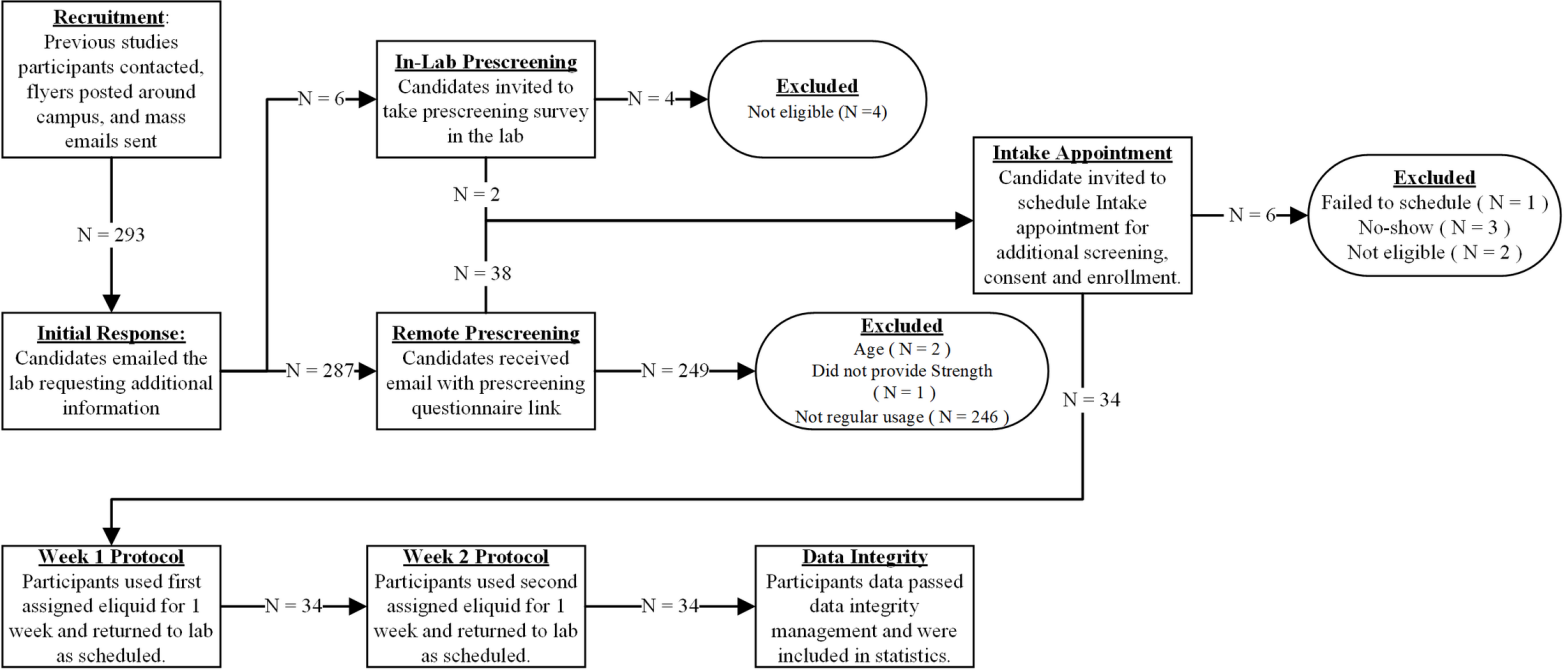
Cohort study flow chart. N = 293 initial responses were received, N = 40 respondents were found eligible and N = 34 participants were enrolled. Data from all enrolled participants were included in the data analysis and are presented in this paper.

A total of N = 293 people responded to the recruitment email and flyers. Most of exclusions (N = 246) were due to not being regular vape pen users. The cohort flow chart, including reasons for exclusion is shown in Fig 3. Of the N = 34 participants completing the final enrollment, 32 were male and 2 were female, ranging in age from 18 to 63 years, with mean age of 23 ± 8 (STD) years. Of the N = 34 participants, 7 indicated a preference for high nicotine strength, 7 for medium nicotine strength and 20 for low nicotine strength, where high, medium and low nicotine levels are defined as 6 mg/ml, 12 mg/ml, 18 mg/ml to be consistent with the PhenX Toolkit [42]. Table 2 gives the detailed cohort information by group. Additional details about the participants’ inhaled tobacco usage are available as S2 Table.”

**Table 2:** Cohort demographics.

Item	Units	Protocol C1: Tobacco / Menthol	Protocol C2: Menthol / Tobacco	Protocol C3: Tobacco / Berry	Protocol C4: Berry / Tobacco
N	[–]	8	9	9	8
Males	[–]	8	9	7	8
Females	[–]	0	0	2	0
Low NIC = 6	[mg/ml]	5	3	8	4
Med NIC = 12	[mg/ml]	2	2	1	2
High NIC = 18	[mg/ml]	1	4	0	2
Min Age	[yrs.]	19	19	18	18
Max Age	[yrs.]	29	63	29	35
Mean Age ± SEM	[yrs.]	24± 1	27± 5	21 ± 1	23 ± 2

Shown are the demographics for the N = 34 participants recruited who were regular electronic vape pen users. Also shown are the participants’ reported regular nicotine levels. Nicotine levels are the reported strengths are categorized by the PhenX protocol PX730301-NicotineContent [42].

All participants who enrolled in the study, completed the study.

### Data integrity analysis

Data integrity analysis indicated no major issues with the wPUM^TM^ monitor performance or monitoring data collected over the two-week period. Condensation and contamination of the monitor which were observed in our previous study with generation 1 e-cigs [38] were not observed with the NJOY^TM^ EVP utilized in the present study. There were some anomalies in the monitoring data collected on Wednesdays (the day of the intake, switching and outtake appointments), which was determined by close inspection of the timestamps associated with each session, to have been caused by participants not starting and stopping their monitoring periods at the same time each week. As a result, some participants’ Wednesday data represented the equivalent of less than one full day while others represented the equivalent of more than one full day. Since the Wednesday data was not a reliable indicator of actual daily use behavior, the decision was made to eliminate the Wednesday data (associated with intake day, switching day, and outtake day) and analyze only data collected from Thursday (the first full day of natural environment use) to the following Tuesday (the last full day of natural environment use) with the same e-liquid flavor, referred to hereafter as the 6-day data set. Some sessions were omitted from the 6-day data set (22 sessions) due to null files, which the investigators attributed to a file read error or a behavior which may be associated with momentary power-on and power-off of the monitor. As a result of the phase 0 data integrity analysis, a total of 873 (93%) of the 895 total sessions recorded during the 6-day monitoring periods were retained for phase 1, phase 2 and phase 3 analysis. Data retained represented two flavors each for all the 34 participants (see Table 3).

**Table 3:** Number of sessions retained as a result of phase 0—data integrity management.

	Berry	Menthol	Tobacco	Grand Total
**Total**	**184**	**268**	**421**	**873**
**T/B Group**	**184**		**147**	**331**
03	6		3	9
04	5		10	15
07	3		0	3
08	8		5	13
11	14		10	24
12	16		6	22
15	8		17	25
16	10		10	20
17	11		6	17
19	3		6	9
20	4		6	10
23	2		10	12
24	12		8	20
27	40		27	67
28	13		4	17
31	6		7	13
32	23		12	35
**T/M Group**		**268**	**274**	**542**
01		16	10	26
02		3	4	7
05		7	9	16
06		41	44	85
09		9	13	22
10		24	19	43
13		3	6	9
14		19	19	38
18		6	4	10
21		9	7	16
22		12	10	22
25		19	16	35
26		33	53	86
29		6	11	17
30		16	4	20
33		20	25	45
34		25	20	45

Shown are the number of session files from the 6-day data set which were retained for each particpant for each flavor, after phase 0 data integrity analysis. Of the sessions recorded in the 6-day data set, only 7% of the data were removed, and were due to null files attributed to a file read error.

### Descriptive statistics

**Fig 4:**
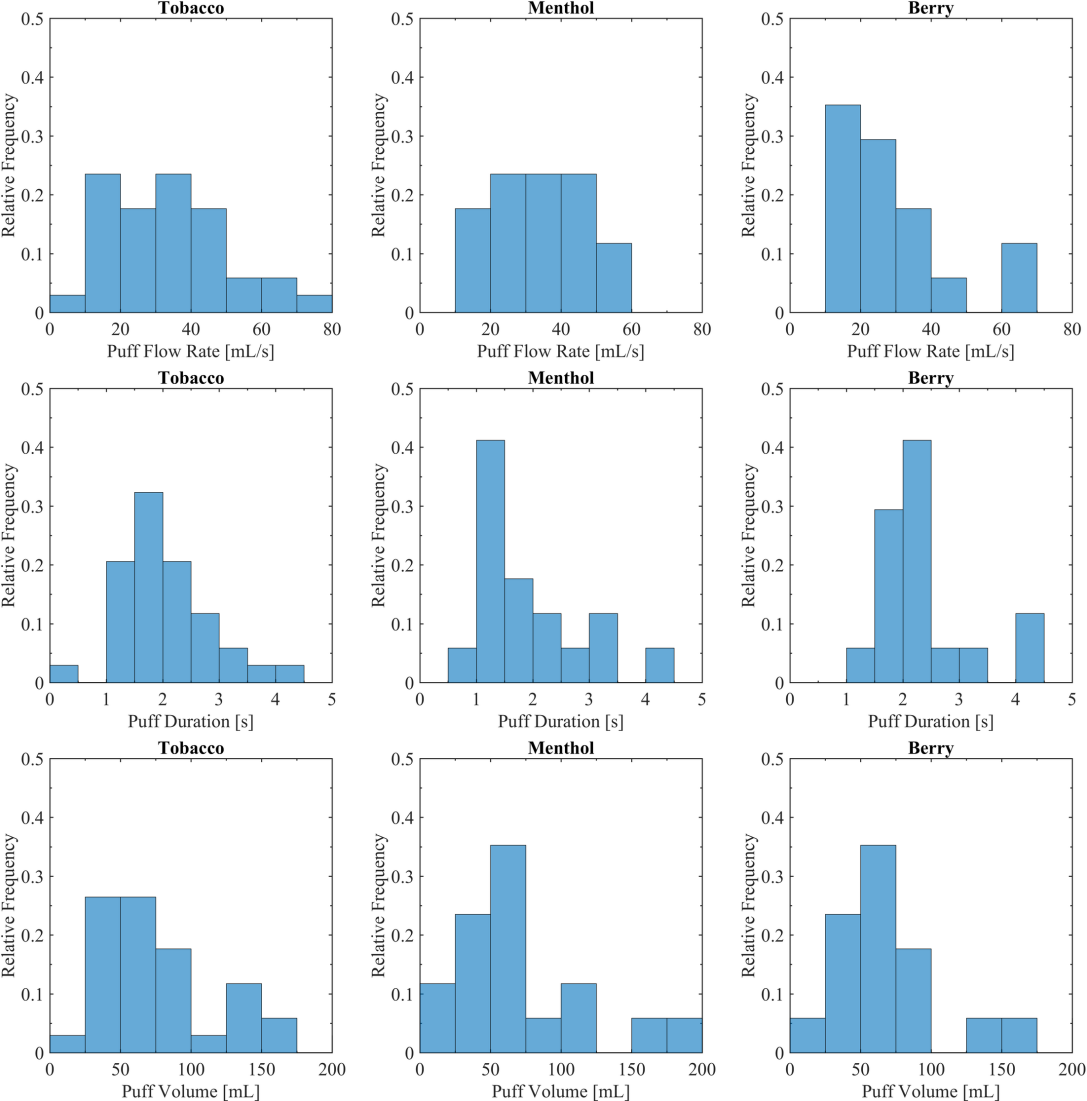
Descriptive cohort statistics for topography behavior. Shown are the histograms illustrating the range of topography behavior characteristics associated with participants assigned to each flavor. The tobacco flavor was used by all 34 subjects for one week, while N = 17 used menthol and N = 17 used berry during the alternate week. Switching order was balanced and randomized.

Topography behavior mean values for each participant were calculated using data from each 6-day period (Thursday to Tuesday). Histograms showing the frequency distribution of participant’s mean topography behavior characteristics are provided in Fig 4 for topography behavior indicators; mean puff flow rate, mean puff duration and mean puff volume. Participant mean puff flow rates ranged from 12.6 ml/s to 72.0 ml/s with a group-mean of 34.8 ml/s for tobacco (N = 37), 34.6 ml/s for menthol (N = 17) and 30.2 ml/s for berry (N = 17). Participant puff durations ranged from 0.8 sec to 4.5 sec with a group-mean of 2.2 for tobacco, 1.9 for menthol and 2.4 for berry. Participant puff volumes ranged from 19.5 ml to 319.4 ml with group-means of 85.4 ml for tobacco, 70.7 ml for menthol and 85.2 ml for berry.

**Fig 5:**
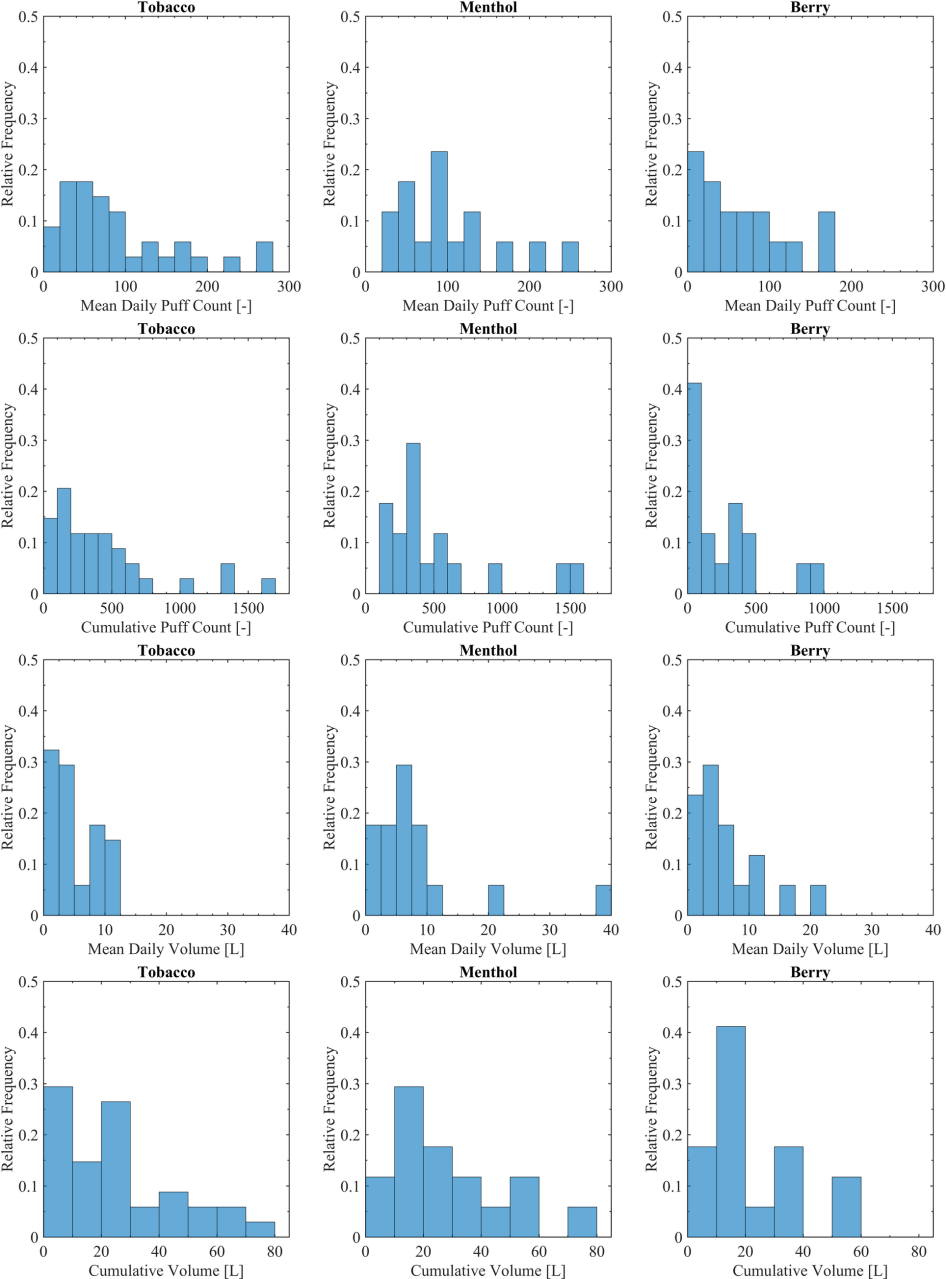
Descriptive cohort statistics for consumption behavior. Shown are the histograms illustrating the range of topography behavior characteristics associated with participants assigned to each flavor. The tobacco flavor was used by all 34 subjects for one week, while N = 17 used menthol and N = 17 used berry for the other week. Switching order as balanced and randomized.

Consumption behavior mean values for each participant were calculated using data from each 6-day period (Thursday to Tuesday). Histograms showing the frequency distribution of participant’s mean consumption behavior characteristics are provided in Fig 5 for consumption behavior indicators; mean daily volume, mean daily puff count, cumulative aerosol volume inhaled and cumulative puff count over the monitoring period. Mean daily volume by participant ranged from 0 ml to 39.7 liters with a group-mean of 6.9 liters for tobacco (N = 37), 7.1 liters for menthol (N = 17) and 4.8 liters for berry (N = 17). Mean daily puff count by participant ranged from 0 puff/day to 315 puffs/day with a group-mean of 92 puffs/day for tobacco, 100 puffs/day for menthol and 74 puffs/day for berry. Cumulative volume by participant (totaled over 6 full days of monitoring) ranged from 0 liters to 133.9 liters with group-means of 31.8 liters for tobacco, 27.1 liters for menthol and 21.5 liters for berry. Cumulative puff count by participant (totaled over the 6 full days of monitoring) ranged from 0 puffs to 1643 puffs with a group-mean of 448 puffs for tobacco, 440 puffs for menthol and 338 puffs for berry.

### Impact of E-liquid flavor on topography and consumption behavior

An ANOVA between groups indicated there was no effect of flavor assignment order on any of the study outcomes (*e*.*g*., for mean flow rate, no difference in condition C1 *vs*. C2, *p = 0*.*981*; and no difference in condition C3 *vs*. C4, *p = 0*.*157*). Therefore, conditions C1 and C2 were grouped as the T/M group and conditions C3 and C4 were grouped as the T/B group for the test of proportions.

**Fig 6:**
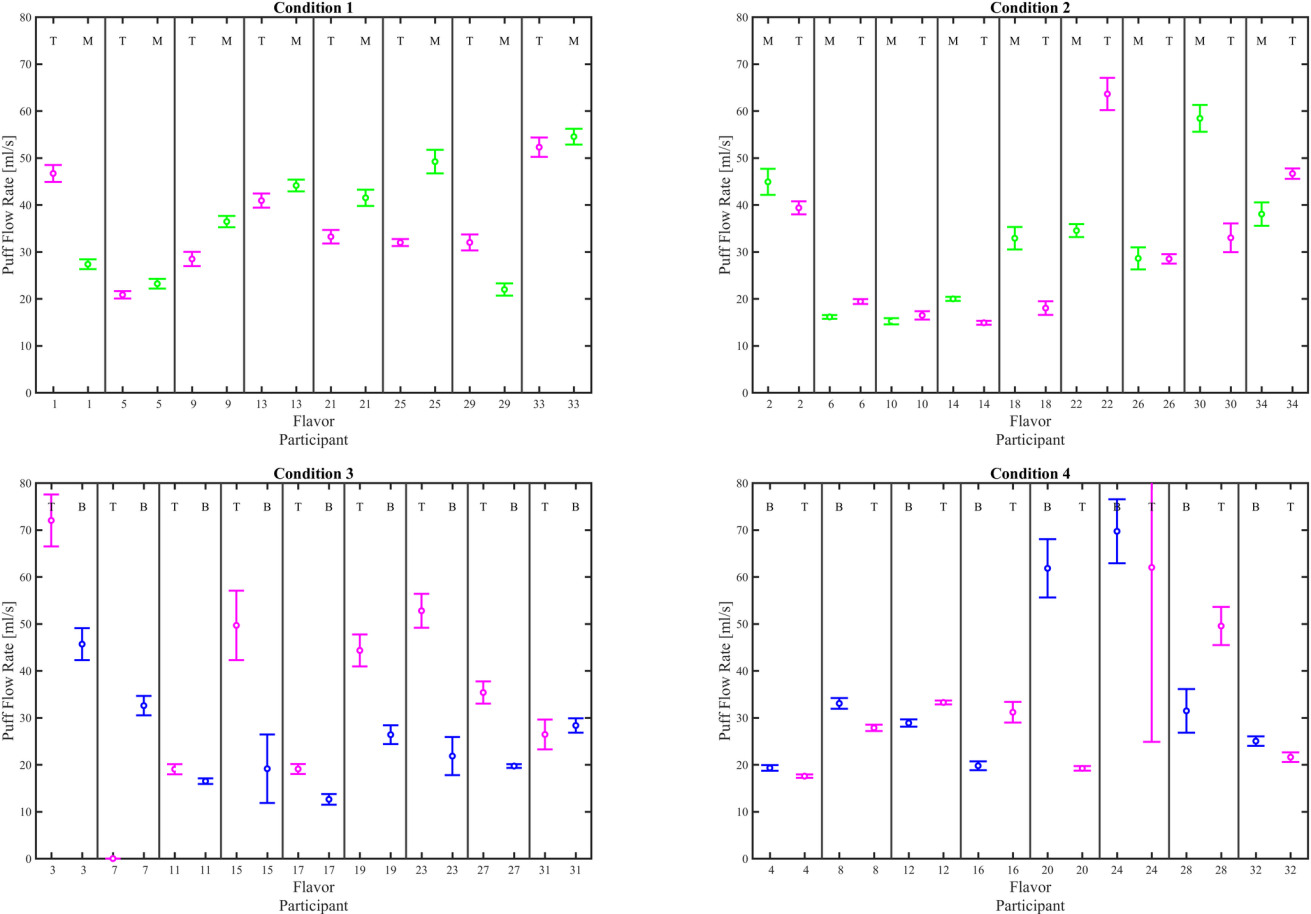
Effect of flavor assignment on mean flow rate. Shown are within-subjects pairwise comparison between flavor for each participant. The left column shows results for participants who were assigned T the first week, and the right column shows results for participants who were assigned T the second week. The top row shows particpants who switched between tobacco and menthol and the bottom row shows participants who switched between tobacco and berry. The mean and 95% CI are computed across all puffs taken by each participant during the 6 day observation period. The flavor for each data set is indicated by a T, M or B at the top of the plot for each participant’s flavor where T = Tobacco, M = Menthol, and B = Berry. Underlying data is available in S6, S7, S8, S9 Datasets.

**Fig 7:**
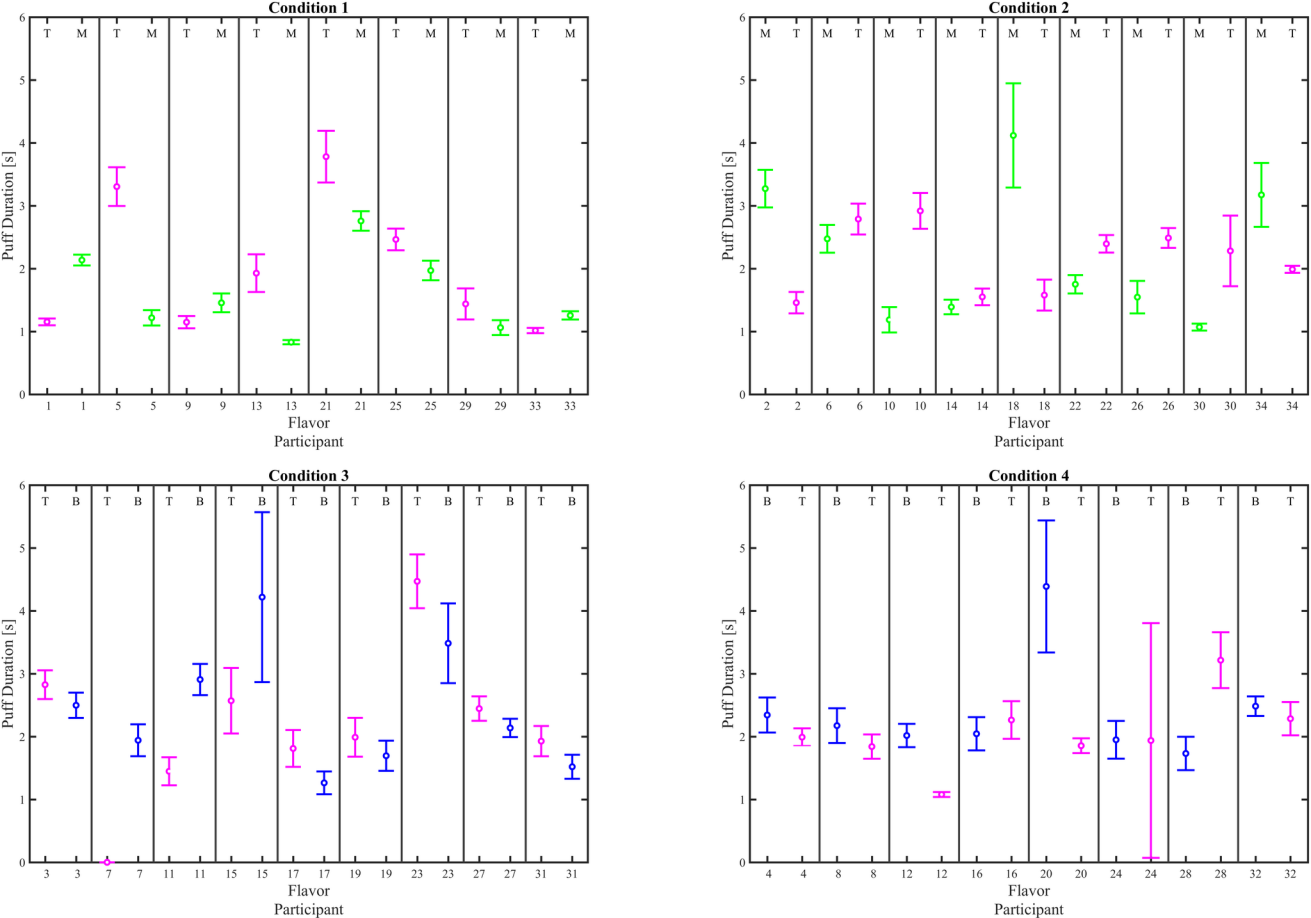
Effect of flavor assignment on mean puff duratio. Shown are within-subjects pairwise comparison between flavor for each participant. The left column shows results for participants who were assigned T the first week, and the right column shows results for participants who were assigned T the second week. The top row shows particpants who switched between tobacco and menthol and the bottom row shows participants who switched between tobacco and berry. The mean and 95% CI are computed across all puffs taken by each participant during the 6 day observation period. The flavor for each data set is indicated by a T, M or B at the top of the plot for each participant’s flavor where T = Tobacco, M = Menthol, and B = Berry. Underlying data is available in S10, S11, S12, S13 Datasets.

**Fig 8:**
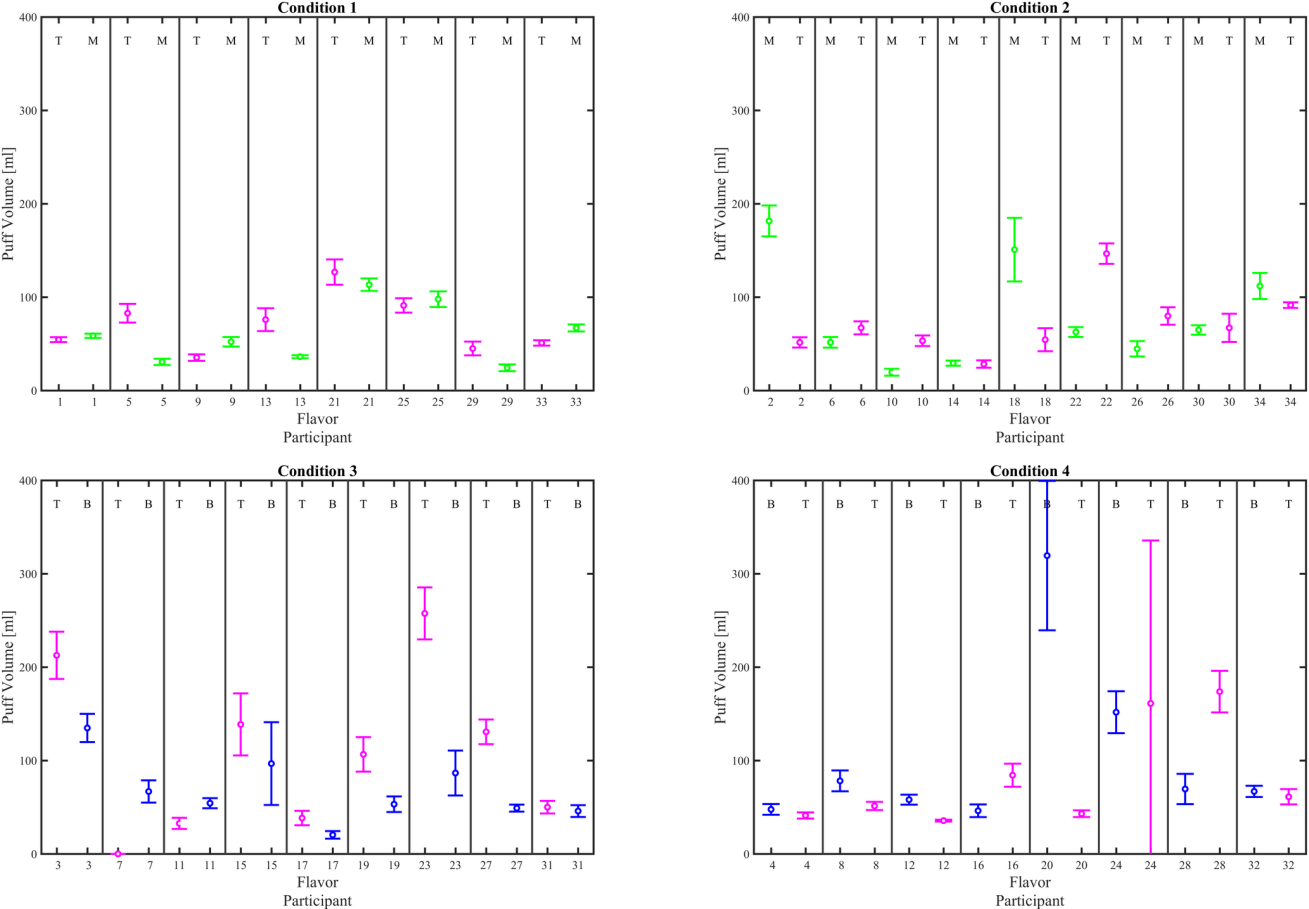
Effect of flavor assignment on mean puff volume. Shown are within-subjects pairwise comparison between flavor for each participant. The left column shows results for participants who were assigned T the first week, and the right column shows results for participants who were assigned T the second week. The top row shows particpants who switched between tobacco and menthol and the bottom row shows participants who switched between tobacco and berry. The mean and 95% CI are computed across all puffs taken by each participant during the 6 day observation period. The flavor for each data set is indicated by a T, M or B at the top of the plot for each participant’s flavor where T = Tobacco, M = Menthol, and B = Berry. Underlying data is available in S14, S15, S16, S17 Datasets.

Interval plots (mean ± 95% CI), given in Figs 6, 7 and 8, illustrate the impact of e-liquid flavor on mean puff flow rate, mean puff duration and mean puff volume, respectively for each participant switching between flavors. 29 of the 34 participants exhibited a significant difference in mean puff flow rate (α_c_ = 0.00147), 23 exhibited a difference in mean puff volume, while 19 exhibited a difference in puff duration, and 4 exhibited a difference in mean puff interval. A test of proportions (α = 0.05) was conducted on the results of the pairwise comparisons, to assess the impact of flavor on topography behavior indicators as shown in Table 4. Differences were found for puff flow rate (*p < 0*.*001* and puff volume (*p = 0*.*012*), but not puff duration (*p = 0*.*196*) or puff interval (*p > 0*.*999*). Given that at least 27 of the 34 participants used a preferred flavor for at least one of their switching conditions (S1 Table), the low p values provide strong evidence that a change in flavor affects puff flow rate and evidence that a flavor change affects puff volume.

**Table 4:** Test of proportions on topography behavior indicators.

Item	Puff Flow Rate [ml/s]	Puff Duration [s]	Puff Volume [ml]	Puff Interval [s]
Total	N = 34	N = 34	N = 34	N = 34
Count (Reject H_0_)	29	19	23	4
Count (Fail to Reject H_0_)	5	15	11	30
Test of Proportions, p Value	<< 0.001	0.196	0.012	> 0.999

Test of proportions for the null hypothesis that “topography behavior is independent of e-liquid flavor.” Topography behavior indicators tested include: mean puff flow rate, mean puff duration, mean puff volume and mean puff interval. Significant relationships are established between e-liquid flavor switching and mean puff flow rate and mean puff volume. Insufficient evidence is provided to establish a relationship beween e-liquid flavor switching and mean puff duration and mean puff interval.

The directionality effect of e-liquid flavor on topography behavior indicators is shown in Table 5. There was no clear directionality in topography indicators. For example, in the T/M cohort, 5 participants had larger flow rates when using T flavor, while 9 had larger flow rates when using M flavor. In the T/B group, 10 participants had larger flow rates when using T flavor, while 5 had larger flow rates when using B flavor.

**Table 5:** Directionality effect of E-liquid flavor on topography behavior indicators.

Item	Puff Flow Rate [ml/s]	Puff Duration [s]	Puff Volume [ml]	Puff Interval [s]
**T/M Cohort**				
Total Count	N = 17	N = 17	N = 17	N = 17
Count (Reject H_0_)	14 (9: T < M) (5: M < T)	14 (9: T < M) (8: M < T)	11 (4: T < M)(7: M < T)	4 (2: T < M) (2: M < T)
Count (Fail to Reject H_0_)	3	3	6	13
Test of Proportions, p Value	0.001	0.001	0.072	0.975
**T/B Cohort**				
Cohort Total Count	N = 17	N = 17	N = 17	N = 17
Count (Reject H_0_)	15 (5: T < B) (10: B < T)	5 (4: T < B) (1: B < T)	12 (5: T < B) (7: B < T)	0
Count (Fail to Reject H_0_)	2	12	5	17
Test of Proportions, p Value	0.000	0.928	0.025	1.000

Shown are the number of participants having significant changes (α = 0.05 FWER, α_c_ = 0.00147 PCER) in their topography between Tobacco (T) and the alternative flavor, either menthol (M) or Berry (B). Also shown are the directionality of the effects (e.g. T > B or B < T) for each cohort and topography behavior. Significant differences were found in puff flow rate for both M and B compared to T and in puff duration M compared to T, but not B compared to T. Difference were evident in puff volume for both M and B compared to T, but only significant for B compared T. No consistent trend in directionality was found.

**Fig 9:**
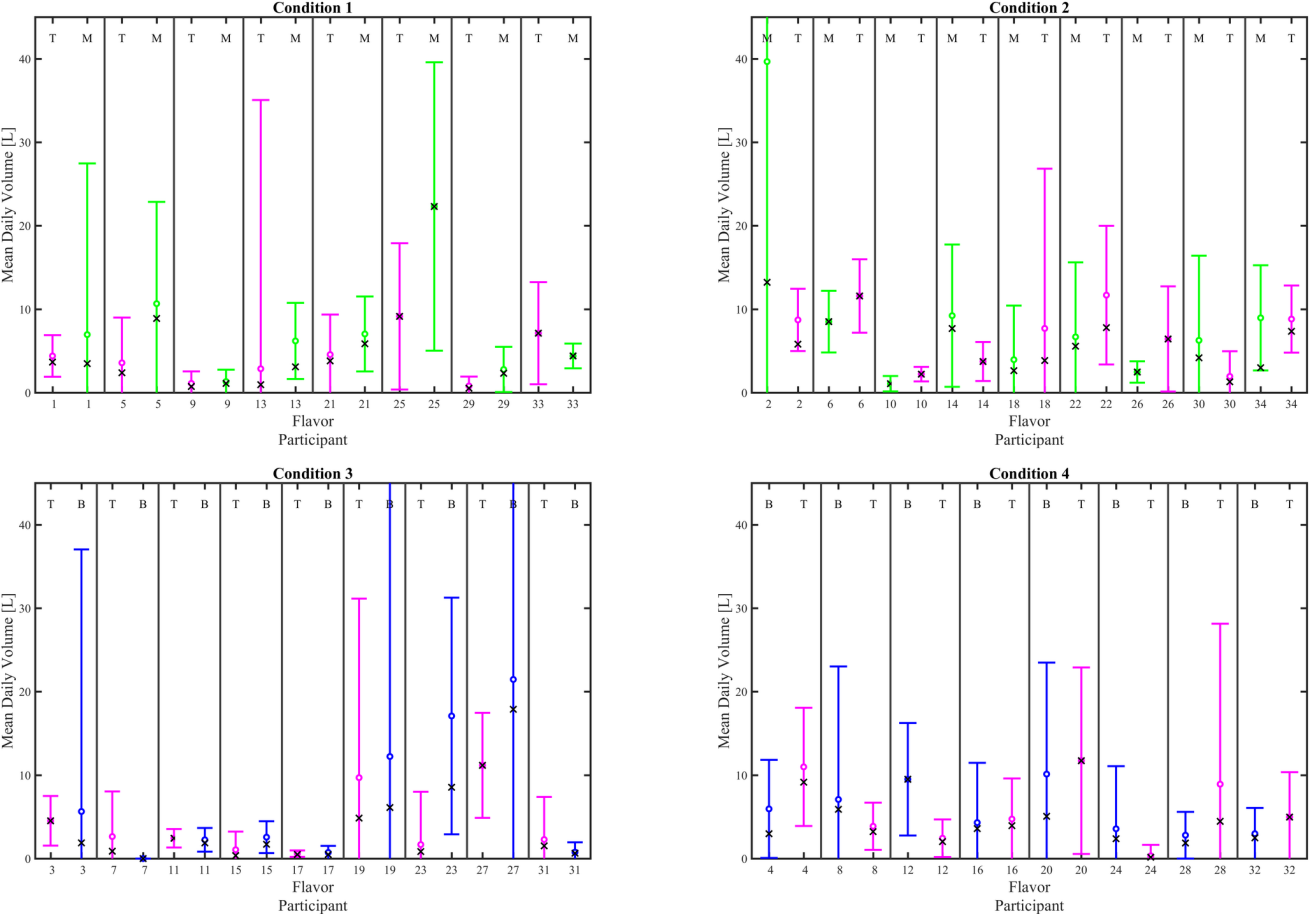
Effect of flavor assignment on average cumulative daily volume. Shown are within-subjects pairwise comparison between flavor for each participant. The left column shows results for participants who were assigned T the first week, and the right column shows results for participants who were assigned T the second week. The top row shows participants who switched between tobacco and menthol and the bottom row shows participants who switched between tobacco and berry. The mean (circle) and 95% CI are computed as the daily average of all days having at least one puffing session during the 6 day observation period. The mean (X) is computed as the cumulative volume divided by 6 days. When the means overlap, the participant exhibited puffing behavior on every day. The flavor for each data set is indicated by a T, M or B at the top of the plot for each participant’s flavor where T = Tobacco, M = Menthol, and B = Berry. Underlying data is available in S18, S19, S20, S21 Datasets.

**Fig 10:**
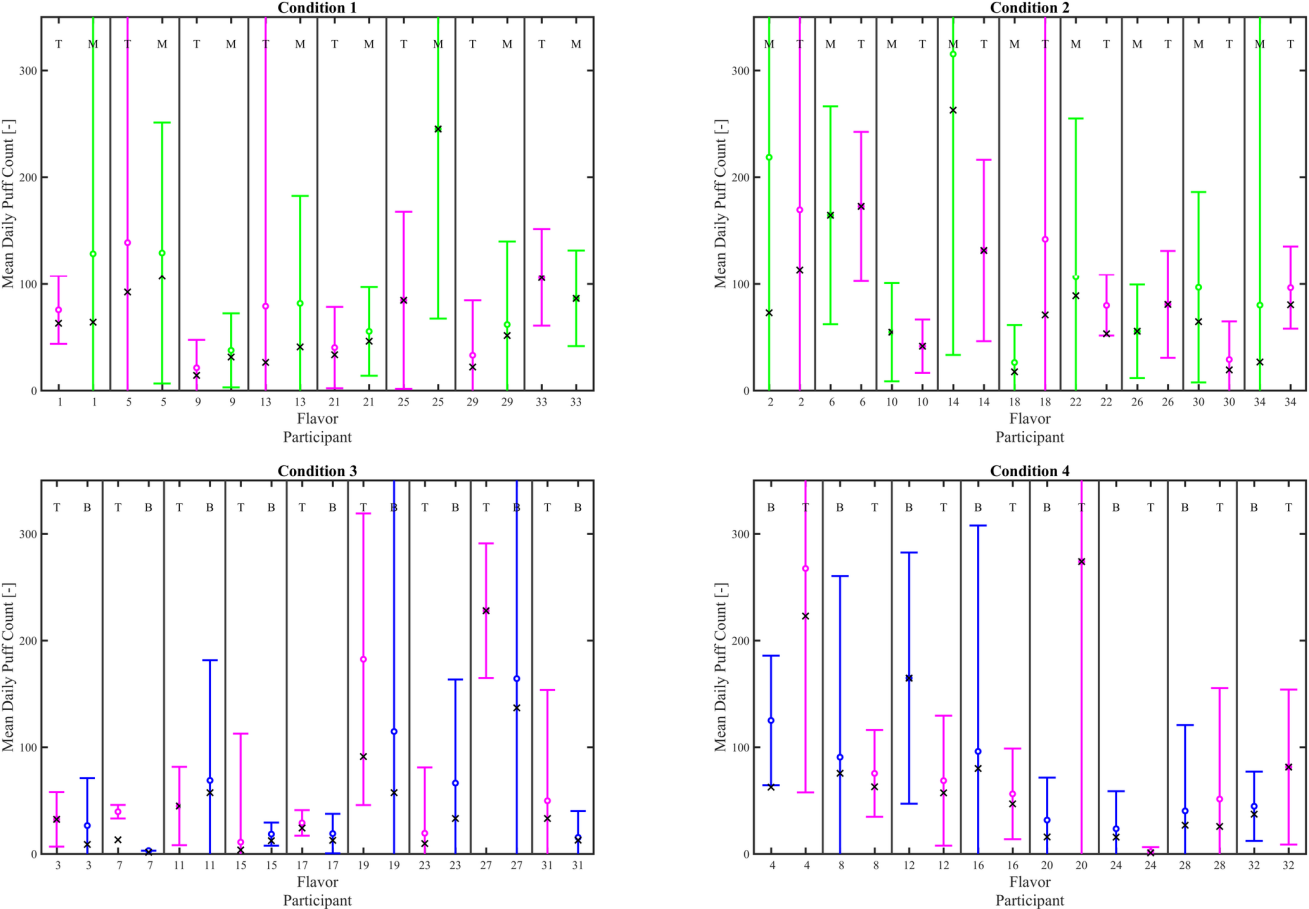
Effect of flavor assignment on average daily puff count. Shown are within-subjects pairwise comparison between flavor for each participant. The left column shows results for participants who were assigned T the first week, and the right column shows results for participants who were assigned T the second week. The top row shows participants who switched between tobacco and menthol and the bottom row shows participants who switched between tobacco and berry. The mean (circle) and 95% CI are computed as the daily average of all days having at least one puffing session during the 6 day observation period. The mean (X) is computed as the cumulative puff count divided by 6 days. When the means overlap, the participant exhibited puffing behavior on every day. The flavor for each data set is indicated by a T, M or B at the top of the plot for each participant’s flavor where T = Tobacco, M = Menthol, and B = Berry. Underlying data is available in S22, S23, S24, S25 Datasets.

Interval plots (mean ± 95% CI), given in Figs 9 and 10 illustrate the impact of e-liquid flavor on mean daily volume and mean daily puff count, respectively for each participant switching between flavors. The mean and 95% CI daily volume and daily puff counts were calculated based on the number of observation days having 1 or more vaping sessions, which varied from 2 to 6. Participant 7 did not vape with Tobacco during the six day observation period and is shown with “zero” characteristics. The mean value for daily puff count and daily puff volume computed as the average across the 6 observation days is illustrated with the “x” symbol for each participant and condition. Comparing each circle with each “x” illustrates the effect of non-puffing days on the consumption behavior of each participant. Fig 9 suggests there may be underlying variations in mean daily cumulative volume as a function of flavor switching, but the limited number of cumulative days of obervations (N = 6) per participant result in relatively large confidence intervals. Similarly, the large confidence intervals on mean daily puff count are also a result of the 6 day observation period. A test of proportions on the within-subjects pairwise comparisons of consumption indicators, shown in Table 6, was insufficent to establish a relationship between e-liquid flavor switching and mean daily volume or mean daily puff count. However, comparing the cumulative puff volumes (over the 6 day period) for each participant by flavor implied some impact of flavor on consumption behavior. As seen in (Table 7), for the T/B cohort, there was no indication of a flavor effect, since the percent consuming more T than B were about equal to those consuming more B than T. However, in the T/M group, there was a clear indication of a flavor effect, because 71% of the T/M cohort participants consumed more T than M, whereas only 29% consumed more M than T.

**Table 6:** Test of proportions on consumption behavior indicators.

Item	Mean Daily Volume [L]	Mean Daily Puff Count [–]
Count (Reject H_0_)	0	0
Count (Fail to Reject H_0_)	34	34
Test of Proportions, p Value	> 0.999	> 0.999

Results shown above indicate that no participants had significant differences (α = 0.05 FWER, α_c_ = 0.00147 PCER) in their mean daily consumption behaviors between tobacco and the alternative flavor. Test of proportions for the null hypothesis that “consumption behavior is independent of e-liquid flavor.” Consumption behavior indicators tested include: mean daily volume and mean daily puff count. Insufficient evidence is provided to establish a relationship beween e-liquid flavor switching and mean daily volume and mean daily puff count.

**Table 7:** Differences in 6-day cumulative volume between flavors.

Flavor	Number (%) consuming larger volumes of each flavor T/M Cohort	Number (%) consuming larger volumes of each flavor T/B Cohort
	N = 17	N = 17
Tobacco	12 (71%)	9 (53%)
Menthol	5 (29%)	
Berry		7 (41%)
No Difference	0 (0%)	1 (6%)

Shown are the number of particpants in each cohort who consumed more of one flavor than the other. For example, 12 participants or 71% of the T/M cohort consumed more tobacco duing their tobacco 6-day period than menthol during their menthol 6-day period. Differences are defined as >50.0% of the total aerosol volume consumed by the participant.

## Discussion and conclusions

This study focused on the relationship between e-liquid flavor and user’s topography and consumption behavior. Topography and consumption behavior were considered separately, since each outcome measure has unique considerations on how the study must be powered and each has different implications on health effect and regulatory policy. Topography describes “how” a user puffs, for example the flow rate, duration and volume of individual puffs, which informs puffing regimes for machine-generated emissions tests [43], whereas consumption describes “how much” aerosol a user inhales over time, which informs risk assessment. While results of this study give some insight into these interactions, more work is needed to address limitations and further explore the impact of e-liquid product characteristics on topography and consumption behavior.

**Fig 11:**
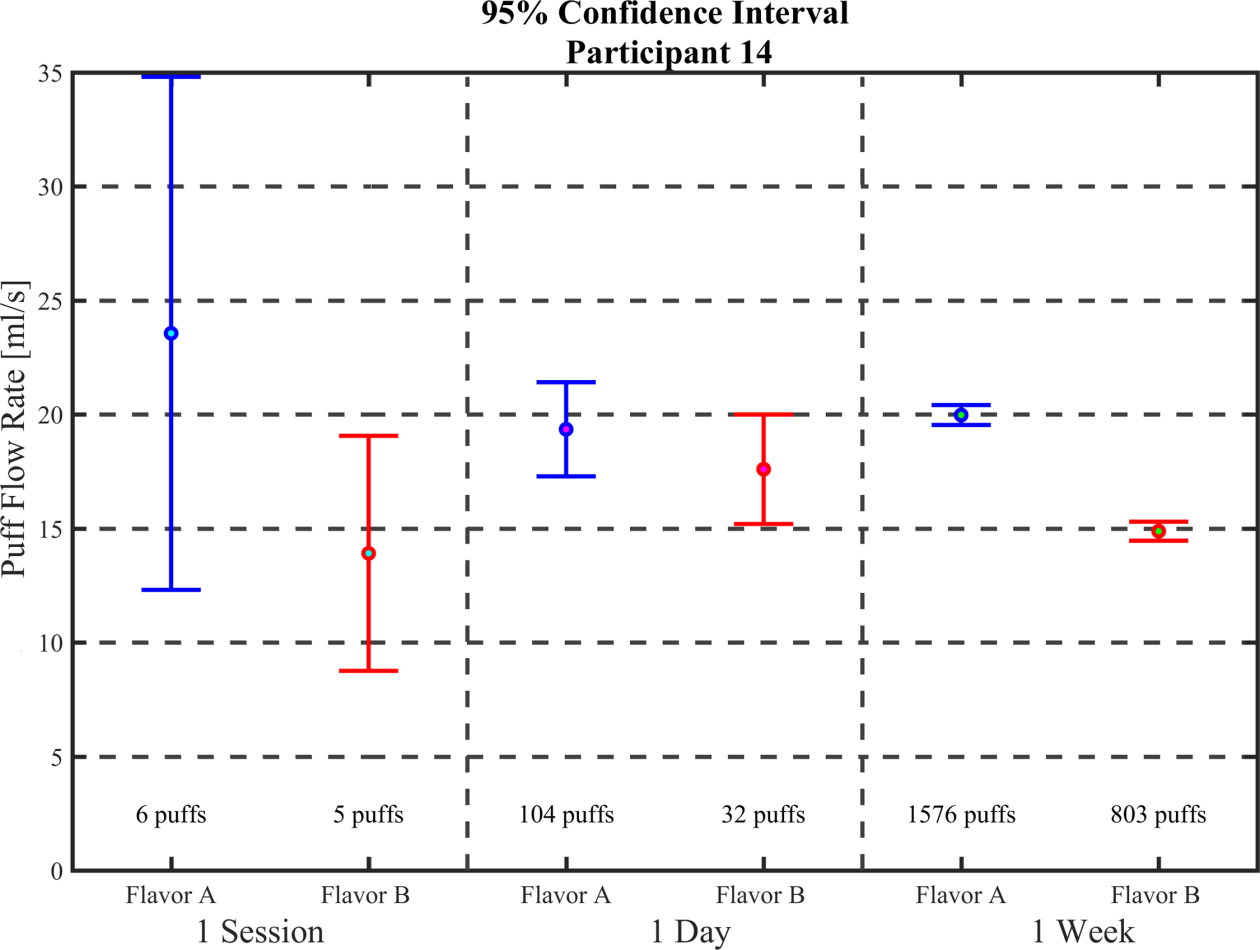
Interval plots for mean puff flow rate for different length monitoring periods. Shown are means and 95% CI for participant 14 from the two-week flavor switching study calculated based on 1 session, 1 day and 1 week of data. Results show that using a monitoring period of less than 1 week would have resulted in a type II error. Underlying data is available in S26 Dataset.

The study provides strong evidence that flavor affects the topography behaviors of mean puff flow rate and mean puff volume, but presents insufficient evidence to support an influence of e-liquid flavor on mean puff duration and mean puff interval. The study was appropriately powered to robustly investigate within-subject pair-wise comparisons, but marginally sufficient to establish between-groups comparisons related to flavor. With total puff counts averaging 525 per week, the 95% CI was sufficiently narrow to detect small differences in affect for a variety of topography behaviors. The interval plot shown in Fig 11 suggests that future studies designed to test the impact of product components on topography behavior should consider monitoring periods of at least 1 week in the natural environment.

The study results were inconclusive regarding the impact of flavor on consumption behavior, primarily due to the low power associated with the six full observation days per condition, to establish a relationship between flavor and cumulative consumption behavior. While the results indicate that an effect may be present, additional observation days are required to establish significance. For example, unlike the topography characteristics which represented the means of over 500 puffs and upwards of 2000 puffs, the consumption characteristics represented means over at most, 6 days per e-liquid flavor. In some cases, the means were calculated over fewer than 6 days due to days having zero sessions. Further, we observed that some participants exhibited wide variability in their daily consumption behavior. While beyond the scope of the current study, we believe that studying the effects of addiction in concert with e-liquid flavors may provide particular insight regarding daily consumption behavior. We choose daily averages as the amount of time for which to characterize consumption, because that seemed to be a reasonable time frame to account for variations due to days of the week [44]. Further studies aimed at detecting differences in consumption behavior as a function of ENDS components should consider longer, perhaps two-week natural environment monitoring periods.

We found significant differences within person on topography behavior but it did not tell a consistent story in terms of how berry or menthol differed from tobacco, expect to say that menthol differs more from tobacco than berry differs from tobacco. These results warrant further study for several reasons [25]. While tobacco company and independent research shows that menthol has sensory properties that could affect use, it is not yet known whether concentration of menthol in e-liquids is a factor that influences puff behavior. Second, even when described or marketed with similar characterizing flavors, e-liquids include varying flavors with unknown effects. For example, tobacco flavor could include a variety of flavorings that vary from one brand to another. Third, cases where there was no consistent trend suggest the difference depends on individual characteristics or preferences. Further study is needed to understand the effects of specific flavorings, flavoring concentrations, and individual-level characteristics on e-cigarette use topography.

Data from the current study was not sufficient to assess the impact of flavor preference on topography or consumption behavior. When asked to rank their favorite flavors among those flavors previously used, the majority of subjects N = 22 (81.5%) reported that of tobacco, candy/fruit, menthol/mint, and “other” flavors, their favorite flavor was candy/fruit. Additional details about the participant’s flavor preferences are available as S1 Table. However, flavor preferences are an important factor in e-cigarette use [21–24] and some flavors, such as menthol, have known analgesic and sensory effects [25]. Indeed, subjects in this study who preferred candy/fruit flavors most frequently reported their reason for their preference as “tastes better.” In contrast, those who preferred menthol/mint flavors most frequently report their reason as “inhaling it feels better.” Further study examining subjects’ usual or preferred flavor as factors when assessing the effect of e-cigarette flavors on topography and consumption is warranted.

The participant’s normal PG:VG ratio was not queried during surveys, and the impact of PG:VG ratio on puffing topography was not considered in this study. The mass ratio of PG:VG was nominally uniform across all conditions in order to reduce the number of variables present upon flavor switching. The ratios measured by NMR were 0.44:0.56 +/- 1% between nicotine strengths for menthol flavor, 0.45:0.55 +/- 1% for berry flavor, and 0.45:0.55 +/- 1% for tobacco flavor. Varying the ratio of PG:VG can give a different sensory experience, which in turn may influence puffing behavior. Further studies are needed to consider the impact of PG:VG ratio on puffing behavior.

Dual-use of cigarettes and e-cigs was permitted and likely occurring during the monitoring protocol for upwards of 56% of the participants based on the intake survey; Of the 34 subjects, N = 15 (44%) were current established smokers and N = 4 (12%) were current non-established smokers *(S2 Table)*. Participants were asked each day during the monitoring period to self-report cigarettes per day, however the response rate was low throughout the study. The highest point (Day 1) had a response rate of 26%. Therefore, we did not include this data in our analysis. However, dual-use of cigarettes and e-cigs is an important factor in assessing the impact of product characteristics on consumption behavior and warrants further study.

The ‘topography behavior’ analysis presented here was focused on simple pair-wise comparisons of changes in topography behavior between switching conditions, similar to the analyses traditionally performed based upon in-lab observations of ad lib smoking and vaping behavior typically lasting 30 minutes or 1 hour. The extended observation period in the natural environment enables much more powerful inferences of the effects of flavor on topography than could be resolved in the lab setting. Furthermore, natural environment observation reduces the confounding effect of participants being asked to use and adapt to a potentially unfamiliar flavor in a short time period. The ‘consumption behavior’ analysis presented here treats each full day of natural environment use as a repeated observation of each participant during each treatment condition.

The ‘consumption behavior’ analysis considers results only for ‘full’ observation days, and excludes the ‘switching day’ of ‘Wednesday’ data, to permit each participant with an opportunity to become familiar with each flavor. Each full observation day (Thurs, Fri, Sat, Sun, Mon, Tues) was considered a single observation for the purposes of estimating consumption variation within participants at each flavor condition and as they switch between conditions.”

Numerous additional studies are enabled by the sophisticated data collection in the natural environment over multiple use sessions. For example, each e-cig device use session could be studied as an observation nested within an individual randomized to one of two nested treatment conditions (flavors), in order to study the evolution of a participant’s topography behavior as they become accustomed to a novel tobacco product. Additionally, the time dependence of participant behavior may be studied to assess variation in topography and consumption behavior differences between week-day and week-end patterns of use. These additional analyses are deferred to a future work.

The emerging literature on ENDS topography clearly demonstrates the need for a standardized topography and consumption monitoring protocols. Variations in the methodologies of published studies, such as the monitoring environment, experienced and non-experienced users, *ad-lib* and prescribed puffing, different measurement devices and different monitoring periods make it difficult draw conclusions from comparisons between published data sets. The current study presented a robust protocol that can be applied to monitor tobacco use topography and consumption behavior in the natural environment and suggests that such a protocol could be adopted by others interested in studying the effects of product characteristics on user behavior.

## Supporting information

S1 Dataset Underlying data for monitor calibration, Fig 1, Calibration date/time: 2016-07-20 16:17. (CSV)

S2 Dataset Underlying data for monitor calibration, Fig 1, Calibration date/time: 2016-07-25 12:40. (CSV)

S3 Dataset Underlying data for monitor calibration, Fig 1, Calibration date/time: 2016-07-27 12:38. (CSV)

S4 Dataset Underlying data for monitor calibration, Fig 1, Calibration date/time: 2016-08-01 12:44. (CSV)

S5 Dataset Underlying data for observed puff flow rate and cumulative volume vs time, Fig 2, Participant 9, Session date/time: 2016-08-19 15:28. (CSV)

S6 Dataset Underlying data for Mean Puff Flow Rate by Participant and Flavor, Fig 6, Switching Condition 1. (DAT)

S7 Dataset Underlying data for Mean Puff Flow Rate by Participant and Flavor, Fig 6, Switching Condition 2. (DAT)

S8 Dataset Underlying data for Mean Puff Flow Rate by Participant and Flavor, Fig 6, Switching Condition 3. (DAT)

S9 Dataset Underlying data for Mean Puff Flow Rate by Participant and Flavor, Fig 6, Switching Condition 4. (DAT)

S10 Dataset Underlying data for Mean Puff Duration by Participant and Flavor, Fig 7, Switching Condition 1. (DAT)

S11 Dataset Underlying data for Mean Puff Duration by Participant and Flavor, Fig 7, Switching Condition 2. (DAT)

S12 Dataset Underlying data for Mean Puff Duration by Participant and Flavor, Fig 7, Switching Condition 3. (DAT)

S13 Dataset Underlying data for Mean Puff Duration by Participant and Flavor, Fig 7, Switching Condition 4. (DAT)

S14 Dataset Underlying data for Mean Puff Volume by Participant and Flavor, Fig 8, Switching Condition 1. (DAT)

S15 Dataset Underlying data for Mean Puff Volume by Participant and Flavor, Fig 8, Switching Condition 2. (DAT)

S16 Dataset Underlying data for Mean Puff Volume by Participant and Flavor, Fig 8, Switching Condition 3. (DAT)

S17 Dataset Underlying data for Mean Puff Volume by Participant and Flavor, Fig 8, Switching Condition 4. (DAT)

S18 Dataset Underlying data for Mean Daily Volume by Participant and Flavor, Fig 9, Switching Condition 1. (DAT)

S19 Dataset Underlying data for Mean Daily Volume by Participant and Flavor, Fig 9, Switching Condition 2. (DAT)

S20 Dataset Underlying data for Mean Daily Volume by Participant and Flavor, Fig 9, Switching Condition 3. (DAT)

S21 Dataset Underlying data for Mean Daily Volume by Participant and Flavor, Fig 9, Switching Condition 4. (DAT)

S22 Dataset Underlying data for Mean Daily Puff Count by Participant and Flavor, Fig 10, Switching Condition 1. (DAT)

S23 Dataset Underlying data for Mean Daily Puff Count by Participant and Flavor, Fig 10, Switching Condition 2. (DAT)

S24 Dataset Underlying data for Mean Daily Puff Count by Participant and Flavor, Fig 10, Switching Condition 3. (DAT)

S25 Dataset Underlying data for Mean Daily Puff Count by Participant and Flavor, Fig 10, Switching Condition 4. (DAT)

S26 Dataset Underlying data for Mean Puff Flow Rate for Participant 14 Switching Flavors, Fig 11, as a function of observation period. (DAT)

S1 Table Cohort Flavor Preferences as Expressed During Intake Survey. (DOCX)

S2 Table Cohort Cigarette Use Patterns as Expressed During Intake Survey. (DOCX)

## References

[1] Food and Drug Administration UDoHaHS. Family Smoking and Tobacco Control Act http://www.fda.gov/TobaccoProducts/GuidanceComplianceRegulatoryInformation/ucm237092.htm2009.

[2] Food and Drug Administration UDoHaHS. Deeming Tobacco Products to be Subject to the Federal Food, Drug and Cosmetic Act, as Amended by the Family Smoking Prevention and Tobacco Control Act. http://www.fda.gov/tobaccoproducts/labeling/rulesregulationsguidance/ucm394909.htm2016.

[3] Food and Drug Administration UDoHaHS. Deeming Tobacco Products to Be Subject to the Federal Food, Drug and Cosmetic Act, as Amended by the Family Smoking Prevention and Tobacco Control Act; Restrictions on the Sale and Distribution of Tobacco Products and Required Warning Statements for Tobacco Products https://www.federalregister.gov/articles/2016/05/2016-10658/deeming-tobacco-produicts-to-be-subject-to-the-federal-food-drug-and-cosmetic-act-as-amended-by-the2016. 27192730

[4] Herning RI, Jones RT, Benowitz NL, Mines AH. How a cigarette is smoked determines blood nicotine levels. Clin Pharmacol Ther. 1983;33:84–90. doi: 0009-9236(83)90568-4 [pii]. . 6848303 10.1038/clpt.1983.12

[5] Herning RI, Jones RT, Bachman J, Mines AH. Puff volume increases when low-nicotine cigarettes are smoked. Br Med J (Clin Res Ed). 1981;283(6285):187–9. ; PubMed Central PMCID: PMCPMC1506678. 6789957 10.1136/bmj.283.6285.187 PMC1506678

[6] Bialous SA, Fox BJ, Glantz SA. Tobacco industry allegations of "illegal lobbying" and state tobacco control. Am J Public Health. 2001;91(1):62–7. ; PubMed Central PMCID: PMCPMC1446513. 11189827 10.2105/ajph.91.1.62 PMC1446513

[7] Arndt J, Vail KE, Cox CR, Goldenberg JL, Piasecki TM, Gibbons FX. The Interactive Effect of Mortality Reminders and Tobacco Craving on Smoking Topography. Health Psychology. 2013;32(5):525–32. doi: 10.1037/a0029201 PubMed PMID: WOS:000318523400007. 23646835 10.1037/a0029201

[8] El Sayed Y, Dalibalta S, Al Tamimi F, editors. Chemical Analysis of The Constituents of Hookah Smoke. International Conference on Environmental Science and Technology; 2015; Rhodes, Greece.

[9] Hatsukami DK, Pickens RW, Svikis DS, Hughes JR. Smoking topography and nicotine blood levels. Addict Behav. 1988;13:91–5. . 3364230 10.1016/0306-4603(88)90031-7

[10] Hatsukami D, Morgan SF, Pickens RW, Hughes JR. Smoking topography in a nonlaboratory environment. Int J Addict. 1987;22(8):719–25. . 3679631 10.3109/10826088709027453

[11] Burling TA, Salvio MA, Seidner AL, Ramsey TG. Cigarette smoking in alcohol and cocaine abusers. Journal of Substance Abuse. 1996;8(4):445–52. doi: 10.1016/s0899-3289(96)90005-x PubMed PMID: WOS:A1996XP43900005. 9058356 10.1016/s0899-3289(96)90005-x

[12] Siegel MB, Tanwar KL, Wood KS. Electronic cigarettes as smoking-cessation tool: results of an online survey. Am J Prev Med. 2011;40 doi: 10.1016/j.amepre.2010.12.006 21406283 10.1016/j.amepre.2010.12.006

[13] Sazonov E, Lopez-Meyer P, Tiffany S. A Wearable Sensor System for Monitoring Cigarette Smoking. Journal of Studies on Alcohol and Drugs. 2013;74(6):956–64. PubMed PMID: WOS:000326768500017. 24172124 10.15288/jsad.2013.74.956 PMC3817052

[14] Brauer LH, Hatsukami D, Hanson K, Shiffman S. Smoking topography in tobacco chippers and dependent smokers. Addictive Behaviors. 1996;21(2):233–8. doi: 10.1016/0306-4603(95)00054-2 PubMed PMID: WOS:A1996UA74300010. 8730526 10.1016/0306-4603(95)00054-2

[15] Ahijevych K, Gillespie J. Nicotine dependence and smoking topography among black and white women. Research in Nursing & Health. 1997; 20(6):505–14. doi: 10.1002/(sici)1098-240x(199712)20:6&lt;505::aid-nur5&gt;3.0.co;2-q PubMed PMID: WOS:A1997YJ63500005. 9397130 10.1002/(sici)1098-240x(199712)20:6<505::aid-nur5>3.0.co;2-q

[16] Aung AT, Pickworth WB, Moolchan ET. History of marijuana use and tobacco smoking topography in tobacco-dependent adolescents. Addictive Behaviors. 2004;29(4):699–706. doi: 10.1016/j.addbeh.2004.02.012 PubMed PMID: WOS:000221679900005. 15135551 10.1016/j.addbeh.2004.02.012

[17] Blank MD, Disharoon S, Eissenberg T. Comparison of methods for measurement of smoking behavior: Mouthpiece-based computerized devices versus direct observation. Nicotine & Tobacco Research. 2009; 11(7):896–903. doi: 10.1093/ntr/ntp083 PubMed PMID: WOS:000267442000017. 19525207 10.1093/ntr/ntp083 PMC2699933

[18] Farsalinos KE, Spyrou A, Stefopoulos C, Tsimopoulou K, Kourkoveli P, Tsiapras D, et al Nicotine absorption from electronic cigarette use: comparison between experienced consumers (vapers) and naive users (smokers) (vol 5, 11269, 2015). Scientific Reports. 2015;5 doi: 10.1038/srep13506 PubMed PMID: WOS:000360638400001. 26082330 10.1038/srep11269 PMC4469966

[19] Lopez AA, Eissenberg T. Science and the evolving electronic cigarette. Preventive Medicine. 2015;80:101–6. doi: 10.1016/j.ypmed.2015.07.006 PubMed PMID: WOS:000362463400019. 26190363 10.1016/j.ypmed.2015.07.006 PMC4592446

[20] Spindle TR, Breland AB, Karaoghlanian NV, Shihadeh AL, Eissenberg T. Preliminary Results of an Examination of Electronic Cigarette User Puff Topography: The Effect of a Mouthpiece-Based Topography Measurement Device on Plasma Nicotine and Subjective Effects. Nicotine & Tobacco Research. 2015; 17(2):142–9. doi: 10.1093/ntr/ntu186 PubMed PMID: WOS:000350142300004. 25239957 10.1093/ntr/ntu186 PMC4838000

[21] Czoli CD, Goniewicz M, Islam T, Kotnowski K, Hammond D. Consumer preferences for electronic cigarettes: results from a discrete choice experiment. Tob Control. 2016;25(e1):e30–6. Epub 2015/10/23. doi: 10.1136/tobaccocontrol-2015-052422 . 26490845 10.1136/tobaccocontrol-2015-052422

[22] Kim H, Lim J, Buehler SS, Brinkman MC, Johnson NM, Wilson L, et al Role of sweet and other flavours in liking and disliking of electronic cigarettes. Tob Control. 2016;25(Suppl 2):ii55–ii61. Epub 2016/11/01. doi: 10.1136/tobaccocontrol-2016-053221 ; PubMed Central PMCID: PMCPMC5489117. 27708124 10.1136/tobaccocontrol-2016-053221 PMC5489117

[23] St Helen G, Dempsey DA, Havel CM, Jacob P 3rd, Benowitz NL. Impact of e-liquid flavors on nicotine intake and pharmacology of e-cigarettes. Drug Alcohol Depend. 2017;178:391–8. Epub 2017/07/14. doi: 10.1016/j.drugalcdep.2017.05.042 ; PubMed Central PMCID: PMCPMC5565733. 28704768 10.1016/j.drugalcdep.2017.05.042 PMC5565733

[24] Barnes AJ, Bono RS, Lester RC, Eissenberg TE, Cobb CO. Effect of Flavors and Modified Risk Messages on E-cigarette Abuse Liability. Tob Regul Sci. 2017;3(4):374–87. Epub 2017/12/06. doi: 10.18001/TRS.3.4.1 ; PubMed Central PMCID: PMCPMC5711435. 29204463 10.18001/TRS.3.4.1 PMC5711435

[25] Lee YO, Glantz SA. Menthol: putting the pieces together. Tob Control. 2011;20 Suppl 2:ii1–7. doi: 10.1136/tc.2011.043604 ; PubMed Central PMCID: PMCPMC3085012. 21504926 10.1136/tc.2011.043604 PMC3085012

[26] Goldenson NI, Kirkpatrick MG, Barrington-Trimis JL, Pang RD, McBeth JF, Pentz MA, et al Effects of sweet flavorings and nicotine on the appeal and sensory properties of e-cigarettes among young adult vapers: Application of a novel methodology. Drug Alcohol Depend. 2016;168:176–80. Epub 2016/10/30. doi: 10.1016/j.drugalcdep.2016.09.014 ; PubMed Central PMCID: PMCPMC5086287. 27676583 10.1016/j.drugalcdep.2016.09.014 PMC5086287

[27] Wagoner KG, Cornacchione J, Wiseman KD, Teal R, Moracco KE, Sutfin EL. E-cigarettes, Hookah Pens and Vapes: Adolescent and Young Adult Perceptions of Electronic Nicotine Delivery Systems. Nicotine Tob Res. 2016;18(10):2006–12. Epub 2016/04/01. doi: 10.1093/ntr/ntw095 ; PubMed Central PMCID: PMCPMC5016844. 27029821 10.1093/ntr/ntw095 PMC5016844

[28] Nonnemaker J, Kim AE, Lee YO, MacMonegle A. Quantifying how smokers value attributes of electronic cigarettes. Tob Control. 2016;25(e1):e37–43. Epub 2015/11/08. doi: 10.1136/tobaccocontrol-2015-052511 . 26546152 10.1136/tobaccocontrol-2015-052511

[29] Hsu G, Sun YJ, Zhu S-H. Evolution of Electronic Cigarette Brands From 2013–2014 to 2016–2017: Analysis of Brand Websites. J Med Internet Res. 2018;20(3):e80 doi: 10.2196/jmir.8550 29530840 10.2196/jmir.8550 PMC5869180

[30] Lawrence D, Cadman B, Hoffman AC. Sensory properties of menthol and smoking topography. Tobacco Induced Diseases. 2011;9(Suppl 1):S3–S. doi: 10.1186/1617-9625-9-S1-S3 PubMed PMID: PMC3102902. 21624149 10.1186/1617-9625-9-S1-S3 PMC3102902

[31] Goniewicz ML, Kuma T, Gawron M, Knysak J, Kosmider L. Nicotine Levels in Electronic Cigarettes. Nicotine & Tobacco Research. 2013;15(1):158–66. doi: 10.1093/ntr/nts103 PubMed PMID: WOS:000312880900020. 22529223 10.1093/ntr/nts103

[32] Behar RZ, Hua M, Talbot P. Puffing Topography and Nicotine Intake of Electronic Cigarette Users. Plos One. 2015;10(2). doi: 10.1371/journal.pone.0117222 PubMed PMID: WOS:000349123100021. 25664463 10.1371/journal.pone.0117222 PMC4321841

[33] Spindle TR, Breland AB, Karaoghlanian NV, Shihadeh AL, Eissenberg T. Preliminary Results of an Examination of Electronic Cigarette User Puff Topography: The Effect of a Mouthpiece-Based Topography Measurement Device on Plasma Nicotine and Subjective Effects. Nicotine & Tobacco Research. 2015; 17(2):142–9. doi: 10.1093/ntr/ntu186 PubMed PMID: WOS:000350142300004. 25239957 10.1093/ntr/ntu186 PMC4838000

[34] Lee YH, Gawron M, Goniewicz ML. Changes in puffing behavior among smokers who switched from tobacco to electronic cigarettes. Addictive Behaviors. 2015;48:1–4. doi: 10.1016/j.addbeh.2015.04.003 PubMed PMID: WOS:000356736900001. 25930009 10.1016/j.addbeh.2015.04.003 PMC4457608

[35] Lopez AA, Hiler MM, Soule EK, Ramôa CP, Karaoghlanian NV, Lipato T, et al Effects of Electronic Cigarette Liquid Nicotine Concentration on Plasma Nicotine and Puff Topography in Tobacco Cigarette Smokers: A Preliminary Report. Nicotine Tob Res. 2016;18(5):720–3. doi: 10.1093/ntr/ntv182 . 26377515 10.1093/ntr/ntv182 PMC5896822

[36] Ossip-Klein D, Megahed N. Low Yield Cigarettes: Risk reduction? Behavioral Medicine Abstracts. 1983;4:73–6.

[37] Robinson RJ, Hensel EC, Morabito PN, Roundtree KA. Electronic Cigarette Topography in the Natural Environment. PLOS ONE. 2015;10(6):e0129296 doi: 10.1371/journal.pone.0129296 26053075 10.1371/journal.pone.0129296 PMC4460076

[38] Robinson RJ, Hensel EC, Roundtree KA, Difrancesco AG, Nonnemaker JM, Lee YO. Week Long Topography Study of Young Adults Using Electronic Cigarettes in Their Natural Environment. PLOS ONE. 2016;11(10):e0164038 doi: 10.1371/journal.pone.0164038 27736944 10.1371/journal.pone.0164038 PMC5063505

[39] Evans SE, Hoffman AC. Electronic cigarettes: abuse liability, topography and subjective effects. Tobacco Control. 2014;23:23–9. doi: 10.1136/tobaccocontrol-2013-051489 PubMed PMID: WOS:000334635400005. 24732159 10.1136/tobaccocontrol-2013-051489 PMC3995256

[40] Robinson DR, Hensel DE, Morabito P, Roundtree K. Electronic Cigarette Topography in the Natural Environment. PLOS ONE 2015. 10.1371/journal.pone.0129296 PMC4460076 26053075

[41] Robinson RJ, Hensel EC, Roundtree KA, Difrancesco AG, Nonnemaker JM, Lee YO. Week Long Topography Study of Young Adults Using Electronic Cigarettes in Their Natural Environment. PLoS ONE. 2016;11(10):31 doi: 10.1371/journal.pone.0164038 27736944 10.1371/journal.pone.0164038 PMC5063505

[42] Hamilton CM, Strader LC, Pratt JG, Maiese D, Hendershot T, Kwok RK, et al The PhenX Toolkit: get the most from your measures. Am J Epidemiol. 2011;174(3):253–60. Epub 2011/07/14. doi: 10.1093/aje/kwr193 ; PubMed Central PMCID: PMCPMC3141081. 21749974 10.1093/aje/kwr193 PMC3141081

[43] Korzun T, Lazurko M, Munhenzva I, Barsanti KC, Huang Y, Jensen RP, et al E-Cigarette Airflow Rate Modulates Toxicant Profiles and Can Lead to Concerning Levels of Solvent Consumption. ACS Omega. 2018;3(1):30–6. doi: 10.1021/acsomega.7b01521 29399647 10.1021/acsomega.7b01521 PMC5793035

[44] Lee YO, Nonnemaker JM, Bradfield B, Hensel EC, Robinson RJ. Examining Daily Electronic Cigarette Puff Topography Among Established and Non-established Cigarette Smokers in their Natural Environment. Nicotine Tob Res. 2017 Epub 2017/10/04. doi: 10.1093/ntr/ntx222 . 29059416 10.1093/ntr/ntx222 PMC6121870

