# Supplementary material for: Effect of e-liquid flavor on electronic cigarette topography and consumption behavior in a 2-week natural environment switching study
Source: PLoS One. 2018 May 2;13(5):e0196640. doi: 10.1371/journal.pone.0196640 (PMC5931659; doi:10.1371/journal.pone.0196640)
Supplement: S1 Table — (DOCX) [file pone.0196640.s027.docx]

*Supplemental Data Table 1 Cohort Flavor Preferences as Expressed During Intake Survey.*

| *Survey Results for Flavor Rankings* | *Observations* | *Number Answered Yes* | *Percent Answered Yes* |
| --- | --- | --- | --- |
| *1st Favorite* |  |  |  |
| *Tobacco* | *27* | *3* | *11%* |
| *Candy/Fruit* | *27* | *22* | *82%* |
| *Menthol/Mint* | *27* | *2* | *7%* |
| *Other* | *0* | *0* | *0%* |
| *2nd Favorite* |  |  |  |
| *Tobacco* | *27* | *6* | *22%* |
| *Candy/Fruit* | *27* | *2* | *7%* |
| *Menthol/Mint* | *27* | *15* | *56%* |
| *Other* | *27* | *4* | *15%* |
| *3rd Favorite* |  |  |  |
| *Tobacco* | *12* | *3* | *25%* |
| *Candy/Fruit* | *12* | *2* | *17%* |
| *Menthol/Mint* | *12* | *4* | *33%* |
| *Other* | *12* | *3* | *25%* |
| *4th Favorite* |  |  |  |
| *Tobacco* | *5* | *3* | *60%* |
| *Candy/Fruit* | *5* | *1* | *20%* |
| *Menthol/Mint* | *5* | *0* | *0%* |
| *Other* | *5* | *1* | *20%* |

*“Shown are the intake survey responses for the N=34 participants recruited who answered survey question “A11 –Flavor Rankings” describing their usual e-liquid flavor preferences. 27 participants provided answers for their “first” and “second” favorite flavors, 12 indicated a “third” favorite flavor and 5 participants indicated a “fourth” favorite flavor. The response to “first” favorite indicates that at least 27 of the 34 participants used a normally preferred flavor consistent with one of the switching condition flavors.”*
