# Supplementary material for: Effect of e-liquid flavor on electronic cigarette topography and consumption behavior in a 2-week natural environment switching study
Source: PLoS One. 2018 May 2;13(5):e0196640. doi: 10.1371/journal.pone.0196640 (PMC5931659; doi:10.1371/journal.pone.0196640)
Supplement: S2 Table — (DOCX) [file pone.0196640.s028.docx]

Supplemental Data Table 2 Cohort Cigarette Use Patterns as Expressed During Intake Survey.

|  | Days smoked in past 30 days | Smoked 100+ cigs in lifetime | Ever smoked cigs | Frequency | Percent | Cumulative Frequency | Cumulative Percent |
| --- | --- | --- | --- | --- | --- | --- | --- |
| Never Smoker | 0 days | No | No | 2 | 6% | 2 | 6% |
| Non-Current Ever Smoker | 0 days | No | Yes | 10 | 29% | 12 | 35% |
| Former Smoker | 0 days | Yes | Yes | 3 | 9% | 15 | 44% |
| Current Non-Established Smoker | 1+ days | No | Yes | 4 | 12% | 19 | 56% |
| Current Established Smoker | 1+ days | Yes | Yes | 15 | 44% | 34 | 100% |

Of the 34 participants, N=25 (74%) reported that they vaped daily. Participants self-reported whether they had ever smoked cigarettes, past 30-day cigarette smoking, and whether they had smoked at least 100 cigarettes in their lifetime. Participants’ smoking status was determined using responses to these items. Established current smokers reported ever smoking, smoking in the past 30 days and smoked 100+ in lifetime. Non-current ever smokers reported ever smoking but did not smoke in the past 30 days and did not smoke 100+ in lifetime. Non-established current smokers reported ever smoking and smoking in the past 30 days but not 100+ in lifetime. Former smokers reported ever smoking and smoked 100+ in lifetime but did not smoke in the past 30-days. Never smokers did not report ever smoking. Of the 34 subjects, N=15 (44%) were current smokers, N=10 (29%) were non-current ever smokers, N=4 (12%) were current non-established smokers, N=3 (9%) were former smokers, and 2 (6%) were never smokers.
